# Genome-Wide Identification and Expression Analysis of OsbZIP09 Target Genes in Rice Reveal Its Mechanism of Controlling Seed Germination

**DOI:** 10.3390/ijms22041661

**Published:** 2021-02-07

**Authors:** Cheng-Chao Zhu, Chu-Xin Wang, Chen-Ya Lu, Jin-Dong Wang, Yu Zhou, Min Xiong, Chang-Quan Zhang, Qiao-Quan Liu, Qian-Feng Li

**Affiliations:** 1 Key Laboratory of Plant Functional Genomics of the Ministry of Education/Jiangsu Key Laboratory of Crop Genomics and Molecular Breeding/Key Laboratory of Crop Genetics and Physiology of Jiangsu Province, College of Agriculture, Yangzhou University, Yangzhou 225009, China; awdrgbnnn@163.com (C.-C.Z.); yzunxywcx@163.com (C.-X.W.); LUCHENYA163@163.com (C.-Y.L.); wangjd1012@163.com (J.-D.W.); 15750556002@163.com (Y.Z.); xiongmin199401@163.com (M.X.); cqzhang@yzu.edu.cn (C.-Q.Z.); qqliu@yzu.edu.cn (Q.-Q.L.); 2 Co-Innovation Center for Modern Production Technology of Grain Crops of Jiangsu Province/Joint International Research Laboratory of Agriculture and Agri-Product Safety of the Ministry of Education, Yangzhou University, Yangzhou 225009, China

**Keywords:** OsbZIP09, preharvest sprouting, ABA, RNA-seq, DAP-seq, seed germination, rice

## Abstract

Seed dormancy and germination are key events in plant development and are critical for crop production, and defects in seed germination or the inappropriate release of seed dormancy cause substantial losses in crop yields. Rice is the staple food for more than half of the world’s population, and preharvest sprouting (PHS) is one of the most severe problems in rice production, due to a low level of seed dormancy, especially under warm and damp conditions. Therefore, PHS leads to yield loss and a decrease in rice quality and vitality. We reveal that mutation of *OsbZIP09* inhibited rice PHS. Analysis of the expression of *OsbZIP09* and its encoded protein sequence and structure indicated that OsbZIP09 is a typical bZIP transcription factor that contains conserved bZIP domains, and its expression is induced by ABA. Moreover, RNA sequencing (RNA-seq) and DNA affinity purification sequencing (DAP-seq) analyses were performed and 52 key direct targets of OsbZIP09 were identified, including *OsLOX2* and *Late Embryogenesis Abundant* (*LEA*) family genes, which are involved in controlling seed germination. Most of these key targets showed consistent changes in expression in response to abscisic acid (ABA) treatment and *OsbZIP09* mutation. The data characterize a number of key target genes that are directly regulated by OsbZIP09 and contribute to revealing the molecular mechanism that underlies how OsbZIP09 controls rice seed germination.

## 1. Introduction

Seeds comprise an extremely important proportion of the world’s diet. Seed dormancy and germination, two distinct physiological processes of seed-bearing plants, are key events in plant development, and are also critical for crop production. Most crops, including rice, were domesticated from wild species that possess a high level of seed dormancy. Cultivated crops germinate easily, which thus ensures high emergence rates after sowing [1]. However, a certain degree of dormancy is critical for seed development and to prevent preharvest sprouting (PHS), which is prevalent in modern crop cultivation and leads to serious agricultural losses. Seed dormancy and germination are precisely controlled by various environmental cues and endogenous stimuli, especially phytohormones [1,2]. Nevertheless, defects in seed germination or the inappropriate release of seed dormancy cause substantial losses in crop yield. Rice is the staple food of more than half of the world’s population. Warm and damp conditions are the major external cause of PHS, which is often encountered during the rice growth season, especially during the maturity stage [3]. Therefore, PHS is one of the most severe problems in rice production, which leads to yield loss and to a decrease in rice quality and vitality [4,5]. Therefore, inhibiting PHS is important in rice breeding programs, as is dissecting its regulatory molecular network and identifying the major genes involved in PHS.

The phytohormones, abscisic acid (ABA) and gibberellins (GAs), are principal regulators that play antagonistic roles in regulating seed dormancy and germination [1,2,6]. GA promotes seed germination, whereas ABA suppresses seed germination, and the balance between ABA and GA determines whether seeds germinate. During seed development, ABA accumulates, which prevents seed germination on the parent plant [7]. Therefore, ABA-deficiency will lead to a high degree of PHS, which extensively reduces crop yield and grain quality [8]. By contrast, enhanced ABA biosynthesis or its reduced catabolism promotes the over-accumulation of ABA, which suppresses seed germination [9,10]. In addition to ABA content, ABA signaling also regulates seed germination. In general, ABA signal transduction is mediated by the core ABA-signaling cascade, which includes ABA receptors, protein phosphatases 2C (PP2Cs), sucrose nonfermenting 1-related protein kinase 2 (SnRK2), and downstream transcription factors [11]. In the presence of ABA, SnRK2s are autophosphorylated and subsequently activate basic leucine zipper (bZIP) transcription factors, which are critical downstream signaling components. These include ABA-responsive element (ABRE)-binding proteins (AREBs), ABRE-binding factors (ABFs), and ABA-INSENSITIVE 5 (ABI5), which further induce the downstream ABA-responsive transcriptional network and plant responses, such as the inhibition of seed germination and enhancement of stress responses [12,13]. 

Transcription factors, such as the above-mentioned bZIP proteins, play key roles in mediating and integrating upstream signals and downstream transcriptional networks, which contribute to the adaptation of plants to the changing environment. bZIP proteins are evolutionarily conserved transcription factors and have been identified in almost all eukaryotes [14]. It is proposed that plant *bZIP* genes originate from four founder genes and that the gene family expanded during evolution [15]. For example, Arabidopsis contains more than 78 *bZIP* genes [16], and 89 are present in rice [17], 96 in *Brachypodium distachyon* [18], 131 in soybean [19], and 247 in rapeseed [20]. 

Despite research on bZIP proteins in rice, most of them have not been cloned or studied. In particular, only several OsbZIPs have been functionally implicated in the regulation of seed germination or PHS. Therefore, the identification and characterization of novel OsbZIPs involved in the modulation of PHS is important. Moreover, further characterization of the genome-wide target gene profiles of OsbZIP proteins will reveal the molecular regulatory network that underlies rice PHS, and is essential to identify key downstream target genes that could specifically regulate rice PHS and be potentially applied in rice breeding programs. Chromatin immunoprecipitation sequencing (ChIP-Seq) is a promising method for determining transcription factor (TF) directly regulated genes in vivo [21]. However, the limitation of this method is the need for high-quality gene-specific antibodies or tagged transgenic lines, which is technically challenging, expensive, and time-consuming. The recently reported DNA affinity purification sequencing (DAP-seq) technique overcomes these limitations by using an in vitro-expressed affinity-tagged TF accompanied by high-throughput sequencing of a genomic DNA library, thereby generating genome-wide target gene maps that reflect both local sequence context and DNA methylation status [22]. Here, we have identified a novel and unique OsbZIP transcription factor, OsbZIP09, mutants of which exhibited inhibited PHS. We first verified the germination properties of the *osbzip09* mutant, and analyzed the *OSbZIP09* expression pattern and the sequence and structure of its encoded protein. We performed RNA-seq analysis to investigate OsbZIP09-regulated transcriptome during seed germination. We also identified the direct genome-wide target genes of OsbZIP09 using DAP-seq. Finally, the data of DAP-seq were analyzed in combination with RNA-seq results to screen key direct target genes of OsbZIP09 that are involved in regulating rice seed germination.

## 2. Results

### 2.1. Mutation of OsbZIP09 Inhibits PHS in Rice and Enhances Rice Sensitivity to ABA during Seed Germination

**Figure 1:**
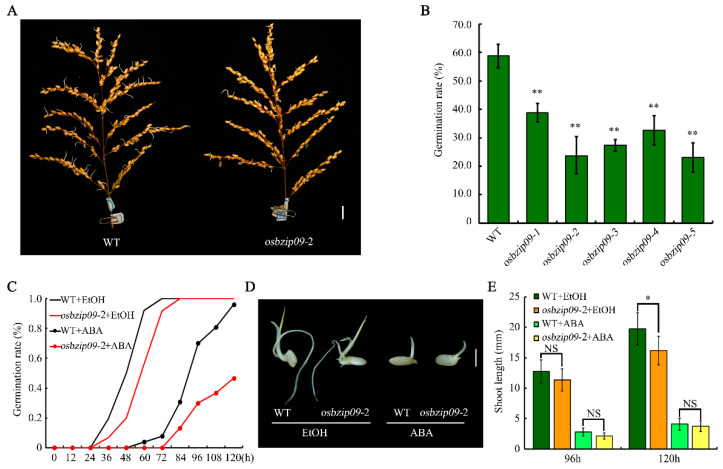
Knock-out of *OsbZIP09* inhibits rice pre-harvest sprouting (PHS). Phenotype (**A**) and germination rate (**B**) of mature rice panicles after 6 d imbibition in water. Scale bar, 2 cm. (**C**) Time-course analysis of seed germination under normal conditions. (**D**) Morphology of germinated seeds 96 h after imbibition (HAI). Scale bar, 0.5 cm. (**E**) Shoot length of germinated seeds 96 and 120 HAI. * *p* < 0.05, ** *p* < 0.01 (*t*-test); NS: not significant.

The PHS phenomenon in rice was severe in Yangzhou in 2018, due to several consecutive rainy days accompanied by high temperatures before the rice harvest. The evaluation of PHS in different rice materials in the field led to the observation that several lines originating from the gene editing of the same target gene, *OsbZIP09*, exhibited less severe PHS than the wild-type control. To analyze the effect of *OsbZIP09* mutation on rice PHS in more detail, we used a growth chamber to mimic high temperature and humidity conditions and evaluated the PHS phenotype of *osbzip09* mutants. The germination rates of all test panicles from the *osbzip09* mutants were notably lower than those of the wild-type control (Figure 1A,B), consistent with the previously observed PHS phenotype in the field. Under normal growth conditions, the germination of *osbzip09* seeds was only slightly delayed (Figure 1C, Figure S1). Because ABA is a key inhibitor of seed germination and bZIP transcription factors play essential roles in ABA signaling, we further evaluated the germination of *osbzip09* mutants in response to ABA. In general, ABA treatment delayed the germination of *osbzip09* and the wild type. However, ABA suppressed the germination of *osbzip09* mutant more severely than that of the controls (Figure 1C, Figure S1), suggesting that *osbzip09* mutant was more sensitive to ABA during seed germination. Analysis of the shoot length of the germinated seeds after 96 h and 120 h indicated that the difference between *osbzip09* and wild type was slight (Figure 1D,E), implying that OsbZIP09 is mainly involved in the regulation of dormancy break and the promotion of seed germination. Based on the result of PHS and seed germination analyses, *osbzip09*-2, which contained a 1-bp insertion in the target site (Figure S2), was selected as a potential line for subsequent RNA-seq and qRT-PCR expression analyses. Further off-target tests indicated that there was no mutation in the potential off-target sites in *osbzip09-*2 (Figure S3), confirming that *osbzip09-*2 was suitable to be a representative line for subsequent expression analyses. 

### 2.2. Analysis of the Phylogenetic Relationships, Protein Sequence and Structure, and Gene Expression of bZIP09

**Figure 2:**
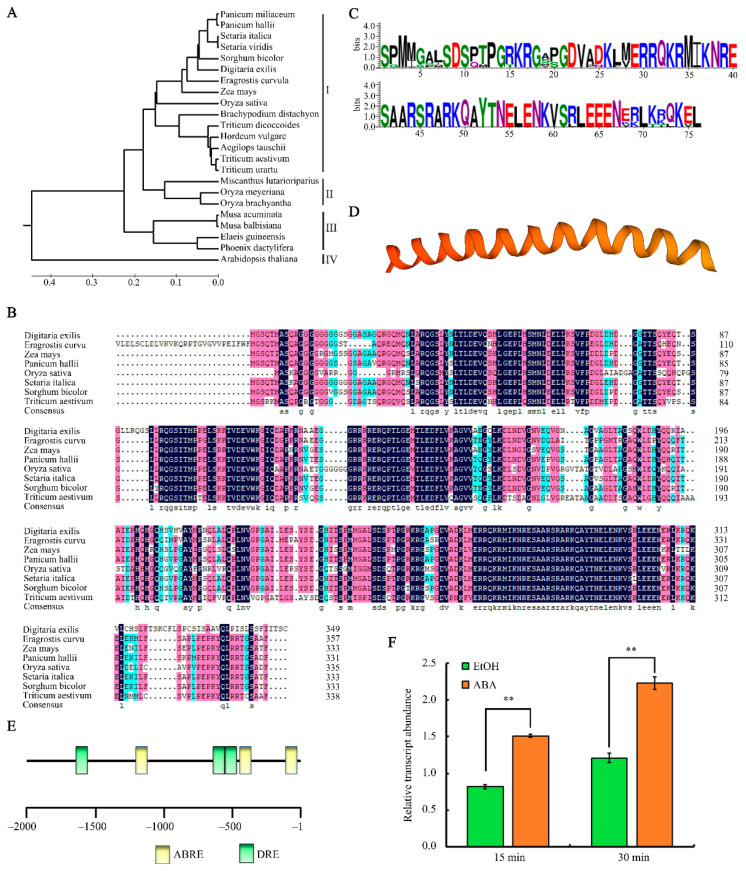
Analysis of the phylogenetic relationships, protein sequence, and structure of bZIP09, and the expression pattern of *bZIP09*. (**A**) Phylogenetic tree of bZIP09 from different plant species. (**B**) Amino-acid sequence alignment of eight bZIP09 homologous proteins. Different homology levels were highlighted in different colors. Navy blue, 100%; pink, between 75% and 100%; light blue, between 50% and 75%. (**C**) Conserved amino-acids in the bZIP09 C terminal region. (**D**) Protein secondary structure analysis of the C terminal region of bZIP09. The conserved domain includes a continuous α-helical structure. (**E**) Analysis of the abscisic acid (ABA)-related *cis*-elements in the *OsbZIP09* promoter. (**F**) Expression of *OsbZIP09* in the wild-type (WT) in response to ABA (5 μM) treatment. ** *p* < 0.01. *ACTIN* served as the internal reference gene for normalization. Values were obtained from three independent experiments. Data are shown as means ± SD.

Because OsbZIP09 is an uncharacterized bZIP transcription factor, we analyzed its amino-acid sequence, protein structure, and gene expression in detail. First, full-length amino-acid sequences of bZIP09 from 23 different plant species were obtained and used to generate a phylogenetic tree. These bZIP09 proteins were classified into four subfamilies, which included 15, 3, 4, and 1 members in groups I, II, III, and IV, respectively (Figure 2A). The bZIP09 amino-acid sequences from eight representative species in group I, including rice, were then aligned, which showed conservation of several regions of the bZIP09 proteins, especially the C terminus where the bZIP domain is located (Figure 2B). Further analyses of the conserved domain at the C terminus, corresponding to 236 to 311 aa in rice, revealed a typical bZIP structure (NX7RX9LX6LX6L), which included a basic DNA-binding region and an adjacent ZIP domain (Figure 2C, Figure S4). Three-dimensional homology modeling of the conserved C terminal region revealed a continuous α-helical structure (Figure 2D). Because osbzip09 was affected in PHS and was also more sensitive to ABA, the *OsbZIP09* promoter (2000 bp upstream of the translation initiation codon ATG) was analyzed for the presence of potential *cis*-acting motifs related to ABA using the PLACE database (http://www.dna.affrc.go.jp/PLACE/, accessed on 18 November 2020). Three ABRE motifs and three DRE motifs (drought-responsive element) were identified (Figure 2E). Expression analysis showed that only 15 min ABA treatment could upregulate the expression of *OsbZIP09* (Figure 2F).

### 2.3. Identification of Genes Co-Regulated by ABA and OsbZIP09 Via RNA-Seq

**Figure 3:**
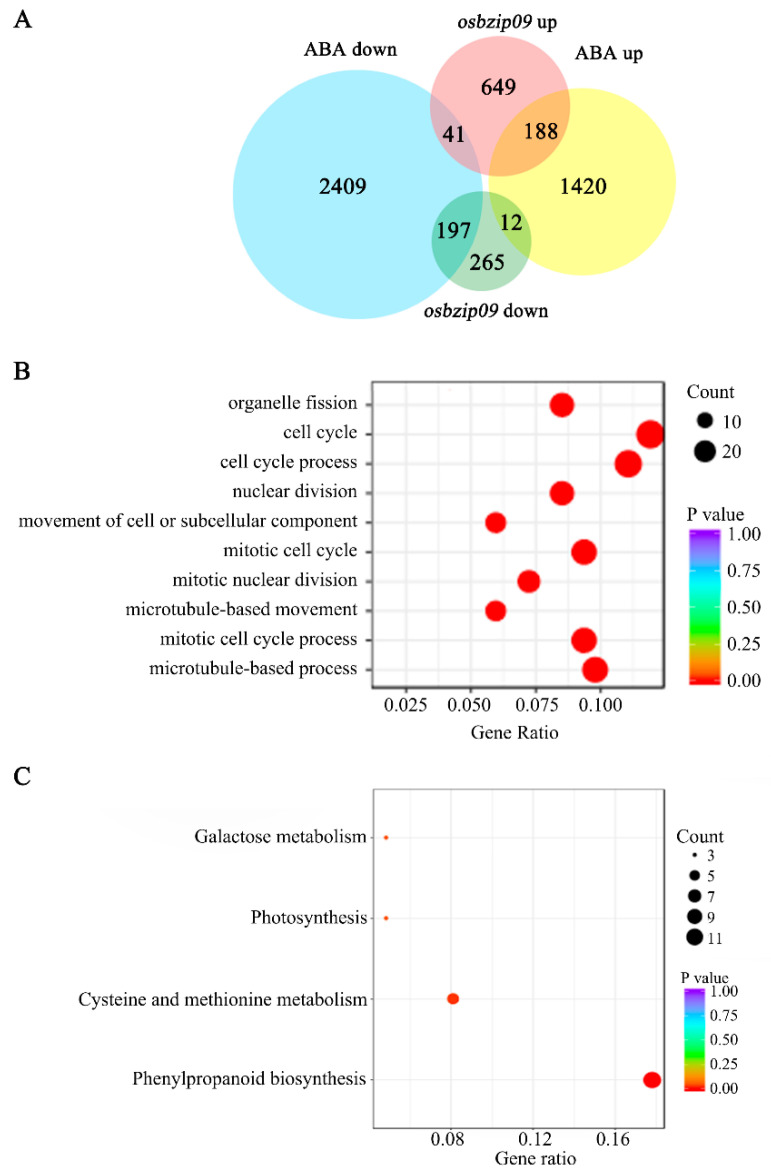
RNA-seq analyses of *osbzip09-*2 and ABA-treated wild-type rice. (**A**) Venn diagrams showing the overlap between genes up- or downregulated by ABA and in *osbzip09-*2. ABA up—ABA-upregulated genes; ABA down—ABA-downregulated genes; *osbzip09* up—upregulated genes in *osbzip09-*2; *osbzip09* down—downregulated genes in *osbzip09-*2. (**B**) Distribution of the top 10 biological process gene ontology (GO) terms for the common differentially expressed genes (DEGs) of ABA treatment and *osbzip09-*2. (**C**) Kyoto Encyclopedia of Genes and Genomes (KEGG) pathways that were enriched among the common DEGs of ABA treatment and *OsbZIP09* mutation.

Because the expression of *OsbZIP09* is induced by ABA and *osbzip09* is more sensitive to ABA during seed germination, OsbZIP09 potentially regulates components of the ABA pathway to coordinate seed germination. To verify this hypothesis, an RNA-seq experiment was performed using germinated seeds (36 h after imbibition (HAI)) from an ABA-treated wild-type and *osbzip09-*2 mutant. More than 6.1 Gb of clean bases were generated and the Q30 value exceeded 89.13% for each sample (Figure S5A). More than 87.94% of reads could be uniquely mapped to the rice genome (Figure S5B). Furthermore, more than 91.89% of the reads mapped to exonic regions (Figure S5C). In total, 4267 differentially expressed genes (DEGs) were identified in response to ABA treatment (fold change >1.5, *p* < 0.05), including 2647 downregulated genes and 1620 upregulated genes (Figure 3A). In *osbzip09-*2, the expression of 1352 genes was altered compared to the wild-type control (fold change > 1.5, *p* < 0.05), 474 of which were downregulated and 878 were upregulated (Figure 3A). Comparison of the genes regulated by ABA and OsbZIP09 indicated that 438 genes were regulated in common (Figure S6), which was almost one-third of all the DEGs in the *osbzip09-*2. Further analysis of these 438 genes showed that 385 showed the same change in expression pattern, with 197 genes being downregulated and 188 genes upregulated in both cases (Figure 3A). This suggests that OsbZIP09 and ABA probably coordinate rice seed germination by coregulating the same set of downstream genes. Gene ontology (GO) analysis of the 438 common targets revealed that genes involved in cell cycle, nuclear decisions, organelle fission, movement of cell, or other components were enriched (Figure 3B). In addition, Kyoto Encyclopedia of Genes and Genomes (KEGG) analysis indicated that the common targets were enriched in pathways of phenylpropanoid biosynthesis, cysteine and methionine metabolism, photosynthesis, and galactose metabolism (Figure 3C). The number of genes enriched in the phenylpropanoid biosynthesis pathway was the largest, and this category is closely related to plant growth and defense [23].

### 2.4. DAP-Seq Identification of the Genes Directly Targeted by OsbZIP09

**Figure 4:**
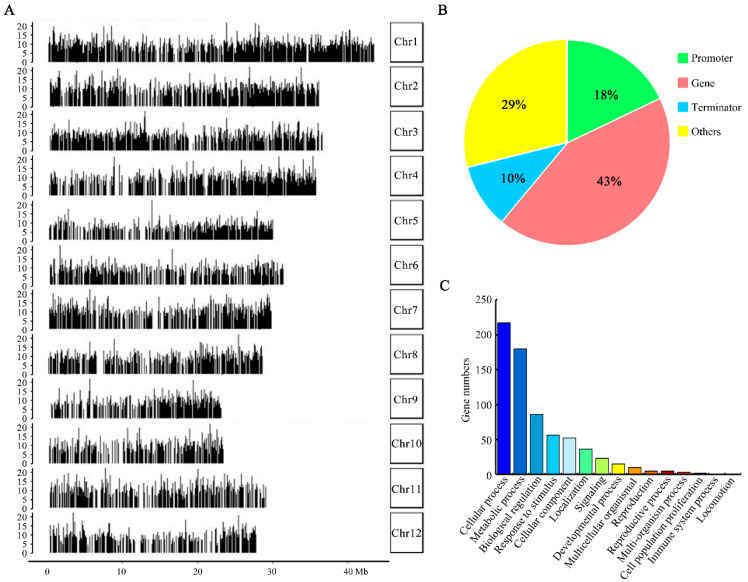
DAP-seq (DNA affinity purification sequencing) analysis of rice OsbZIP09. (**A**) Distribution of OsbZIP09-binding sites along the twelve chromosomes of rice. (**B**) Distribution of OsbZIP09-binding sites in genic and intergenic regions. (**C**) Biological process categorization of OsbZIP09-regulated target genes using gene ontology (GO) analysis.

Because OsbZIP09 is a novel transcription factor in the regulation of seed dormancy and germination, and several DEGs were identified from RNA-seq analysis, one appropriate strategy to further investigate the underlying mechanism underlying OsbZIP09 function was to identify its direct target genes. Therefore, DAP-sequencing [22] was performed for this purpose. The OsbZIP09-binding sites were analyzed using MACS2 [24]. Peaks that were located 2 kb upstream from the transcription start site (TSS) or 2 kb downstream from the transcription termination site (TTS) were considered to bind to the *OsbZIP09* promoter and terminator regions, respectively. In total 7752 binding sites were identified across the whole rice genome (Figure 4A). Among these identified peaks, approximately 18% (1396) were located within the promoter region (2 kb upstream from the TSS), corresponding to 1354 genes (Figure 4B), and a specific core binding motif (CACGTG/C) for OsbZIP09-binding was also revealed. Further gene ontology (GO) analysis indicated that these genes could be assigned into 15 different groups according to their biological processes (Figure 4C). Among these groups, genes belonging to ‘cellular process’ and ‘metabolic process’ constituted the two largest groups and accounted for 36.5% and 28.8% of the total analyzed genes, respectively.

### 2.5. Identification and Analysis of the Core Direct Target Genes of OsbZIP09

**Figure 5:**
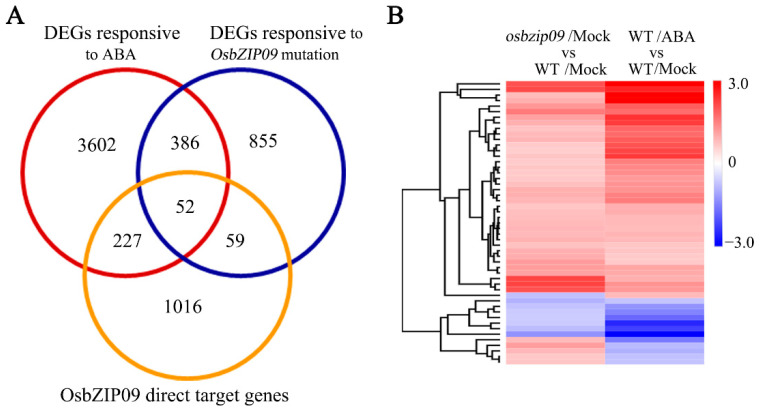
Identification and analysis of OsbZIP09 direct target genes responsive to ABA. (**A**) Venn diagrams showing the overlapping genes between the RNA-seq and DAP-seq data. (**B**) Hierarchical clustering analysis of the 52 common target genes.

On the basis of the results from the RNA-seq analysis, 438 common targets of ABA and *OsbZIP09* were identified, and approximately 90% of these showed the same qualitative change in expression. Comparison of RNA-seq and DAP-seq data could aid the identification of the direct targets of OsbZIP09 that are involved in the regulating of rice seed germination. The comparison identified 52 common gene targets (Figure 5A). According to the RNA-seq expression data, these common genes were classified into different cluster groups by hierarchical clustering analysis (Figure 5B). Notably, 88.9% of the targets shared the same pattern of gene expression change, and 73.1% of the genes were upregulated in response to ABA treatment and in o*sbzip09-*2 mutant. Next, we divided all the 52 genes into three groups according to their mode of expression change. All the information relating to their expression, description, and OsbZIP09-binding site in their promoter is listed in Table 1, Table 2 and Table 3. Table 1 lists the 38 genes whose expression was upregulated in response to ABA and in *osbzip09-*2. These included two genes that encoded F-box proteins, two *OsbZIP* genes, two OsPP2C phosphatase-encoding genes, six *LEAs,* and some other genes. Among these, *LEA* genes constituted the greatest proportion of the common targets and the expression of all *LEA* genes increased in response to ABA or in *osbzip09-*2. Moreover, the change in expression change of *LEA* genes in *osbzip09-*2 was about 1.5- to 2.0-fold of that in the wild-type control, whereas they were expressed about 2.3- to 8.3-fold more highly in the ABA-treated wild-type plants than in the mock-treated wild type, suggesting that loss of OsbZIP09 could only partially mimic the effect of ABA treatment, which is consistent with the fact that several other transcription factors in the ABA pathway also modulate the expression of *LEAs*. The *LEA* genes were considered to be reliable ABA-responsive genes and they are often involved in various stress responses. Table 2 lists eight genes whose expression decreased in response to ABA and in the *osbzip09-*2 mutant, including *DLT* (*DWARF AND LOW-TILLERING*) and *OsLOX2*, which are involved in brassinosteroid (BR) signaling and the oxidation of polyunsaturated fatty acids (PUFAs), respectively. In addition, several genes encoding protochlorophyllide oxidoreductase, nucleoside phosphorylase, chromomethyltransferase, calvin cycle protein, and receptor-like protein kinase were also downregulated.

### 2.6. Expression Analysis and Validation of the Representative Target Genes of OsbZIP09

**Figure 6:**
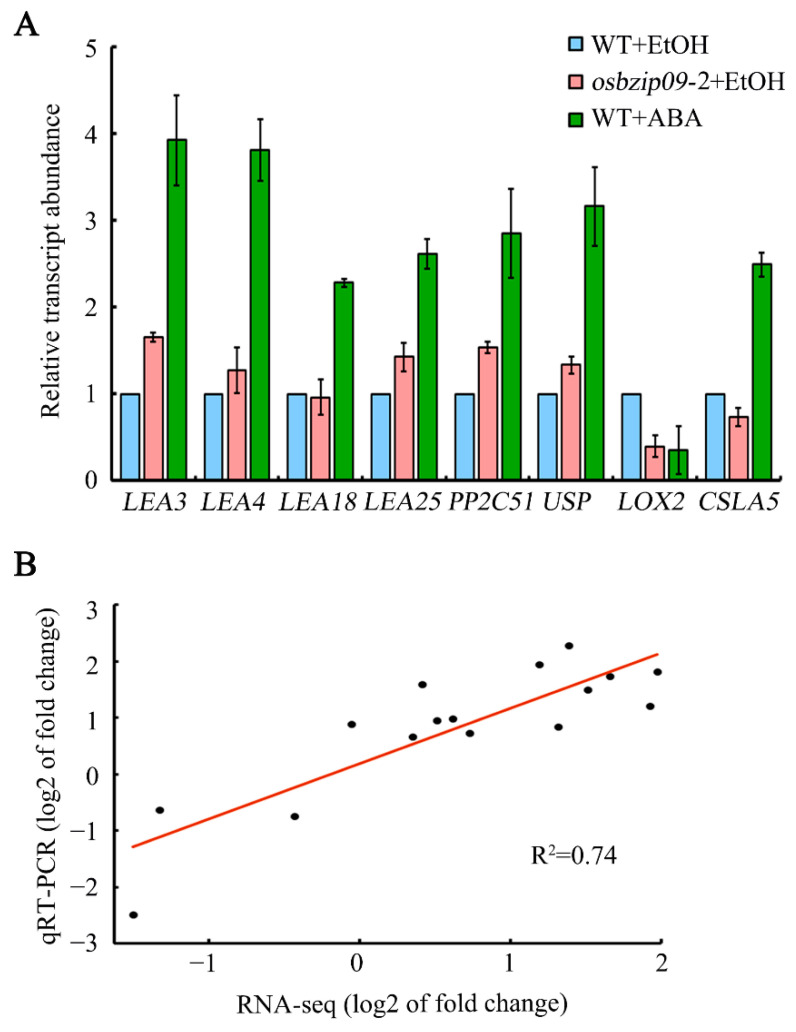
Validation of RNA-seq data for the representative common targets by qRT-PCR. (**A**) qRT-PCR for selected eight representative common targets. (**B**) Correlation of gene expression between qRT-PCR and RNA-seq data. Fold-change values were log_2_ transformed.

To validate further the quality of the RNA-seq data, eight representative genes were selected from the 52 key targets for qRT-PCR, including six genes from Table 1, *LOX2* (Table 2), and *CSLA5* (Table 3). The expression of seven out of the eight genes was consistent with the RNA-seq data (Figure 6A). i.e., the expression of *LEA3*, *LEA4*, *LEA25*, *PP2C51* and *USP* increased in both *osbzip09-2* and ABA-treated wild type, whereas that of *LOX2* decreased in both samples, and the expression of *CSLA5* decreased in response to ABA treatment and increased in *osbzip09-*2 mutant. Only the expression of *LEA18* differed slightly from the RNA-seq data in *osbzip09-*2 mutant. Correlation analysis of the gene expression between qRT-PCR and RNA-seq data indicated that the values were consistent (correlation coefficient *R^2^* = 0.74), confirming the accuracy of the RNA-seq data (Figure 6B). 

### 2.7. Expression Analysis of the Representative Target Genes Regulated by OsbZIP09 and ABA

**Figure 7:**
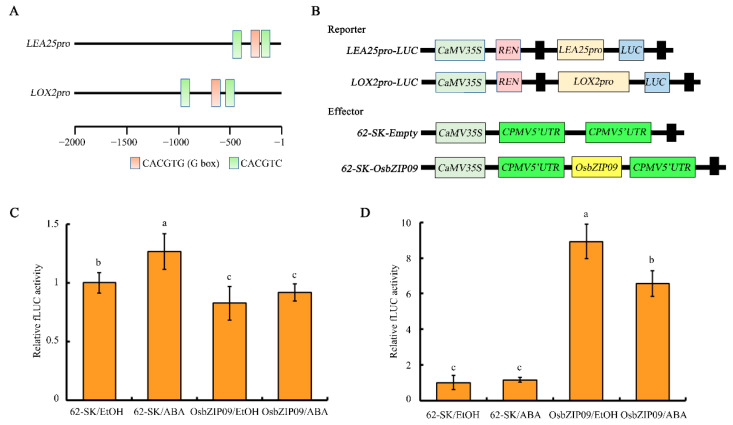
OsbZIP09 controls seed germination by suppressing the expression of *Late Embryogenesis Abundant* (*LEA*) family genes and enhancing the expression of *OsLOX2*. (**A**) OsbZIP09-binding motif analysis in the promoter of *LEA25* and *LOX2*, respectively. (**B**) Schematic depiction of the reporter and effector constructs used in the dual-luciferase reporter assay. (**C**,**D**) OsbZIP09 suppresses the promoter activity of *LEA25* but activates the promoter activity of *LOX2*. Data are shown as means ± SD (*n* = 5). Statistically significant differences at *p* < 0.05 are indicated by different letters.

Because the expression of up to six *LEA* genes was upregulated by ABA treatment and in *osbzip09-*2, *LEA25* was selected as a representative gene for further analysis, and was also identified by previous proteomic study of rice seed germination [25]. Moreover, *LOX2*, which was transcriptionally downregulated in this study and is reported to promote germination [26], was also selected for subsequent assay. Sequence analysis revealed that a conserved CACGTG motif and two CACGTC motifs were identified in the promoters of *LEA25* and *LOX2* (Figure 7A). To verify further the transcriptional regulation of *LEA25* and *LOX2* by OsbZIP09 and ABA, the dual luciferase system was used. The promoters of *LEA25* and *LOX2* were amplified (1.15 kb and 2.05 kb, respectively) and cloned into the pGreen II0800-LUC vector (Figure 7B). In the absence of OsbZIP09, ABA treatment slightly induced the transcription of *LEA25*. OsbZIP09 suppressed the expression of *LEA25* either with or without ABA (Figure 7C), and OsbZIP09 promoted *LOX2* transcription (Figure 7D). Further ABA treatment slightly attenuated the promotive effect of OsbZIP09 on *LOX2* transcription (Figure 7D). Therefore, the direct transcriptional modulation of downstream target genes is an important molecular mechanism for the OsbZIP09-mediated regulation of rice germination.

## 3. Discussion

Pre-harvest sprouting often causes severe losses in grain yield, quality, and germinability in cereal crops, including rice, wheat, barley, and maize [3,27,28]. Diverse endogenous and environmental factors are involved in the regulation of PHS, including humidity, temperature, light, and phytohormones [1,29]. Among these, ABA and GA are the major endogenous determinants that play antagonistic roles in regulating PHS. ABA is critical in the maintenance of seed dormancy, which involves the delay or prevention of seed germination and PHS. Therefore, most PHS in crops is directly or indirectly related to ABA. Many efforts have been made to inhibit crop PHS, including improving growth and cultivation conditions, spraying chemicals, and producing new varieties that are PHS resistant. In general, breeding PHS-resistant crops is the most efficient and environmentally friendly strategy, and this relies on the identification and characterization of the key genes involved in seed dormancy or germination. However, most progress in identifying and characterizing genes has been made in the model plant Arabidopsis. Although many quantitative trait loci (QTLs) or genetic loci associated with PHS have been isolated in rice [30,31,32,33,34], the number of cloned genes is limited and the molecular mechanism that underlies PHS remains elusive. Most of the reported genes relate to ABA metabolism, such as *OsABA1*, *OsPLA3*, *PHS1* to *PHS4*, and *OsCNX6* [5,27,35,36]. Recently, several studies have revealed some regulatory modules involved in PHS. For example, mutation of *PHS8*/*ISA1* enhanced the accumulation of sugars in rice endosperm, thus reducing ABA sensitivity via suppressing the expression of *OsABI3* and *OsABI5* [37]. This suggests that the endosperm sugar content has dual roles in modulating seed dormancy and germination, both as an energy source and as an inhibitor of ABA signaling. Moreover, *PHS9* encodes a CC-type glutaredoxin that directly interacts with OsGAP, an interaction partner of the ABA receptor OsRCAR1. Both PHS9 and OsGAP are negative regulators of ABA signaling and regulate seed germination via integrating signaling between reactive oxygen species (ROS) and ABA [38]. It has also been demonstrated that miR156 is also involved in the regulation of rice PHS. Mutation of mir156 releases its suppression of *Ideal Plant Architecture 1* (*IPA1*), which negatively regulates GA signaling, thus enhancing seed dormancy [39]. Importantly, this study showed that mutation of a specific miR156 could suppress PHS without compromising rice productivity. 

The ABA signaling pathway requires transcription factors to modulate the downstream transcriptional network and consequently ABA-triggered plant responses, such as the suppression of seed germination. The ABA signaling pathway mainly relies on OsbZIP transcription factors, including AREBs, ABFs, and ABI5. Among these, ABI5 plays a key role in suppressing ABA-mediated seed germination and post-germination growth in a number of plant species, such as Arabidopsis [13,40,41], rice [42], wheat [43,44], barley [45], and Sorghum [46]. In addition to the roles of these classical bZIP transcriptional factors in the ABA pathway, several other bZIP members are also involved in the ABA-mediated regulation of seed dormancy and germination in rice. For example, mutation of *OsABF2*/*SsbZIP46* decreased the sensitivity of rice to high levels of ABA at germination and post-germination growth stages [47]. OsbZIP23 directly interacts with MOTHER OF FT AND TFL 2 (OsMFT2), a negative regulator of rice seed germination, and *OsbZIP23* overexpression restores the PHS phenotype of *osmft2* knock-out lines [48]. Moreover, OsbZIP72 is activated by ABA and directly binds to the *Allene Oxide Cyclase* (*AOC*) promoter and enhances its transcription. This subsequently increases the content of endogenous JA and represses seed germination [49]. Furthermore, OsbZIP75 directly binds to the promoter of *DELAY OF GERMINATION 1* (*DOG1*) and promotes the accumulation of OsDOG1L-3 and inhibits seed germination [50]. However, all the above-mentioned OsbZIPs negatively regulate rice germination. In this study, we demonstrated that OsbZIP09 positively promotes seed germination and its mutation inhibits rice PHS and slightly delays seed germination. Moreover, RNA-seq analysis showed that many genes were coregulated by ABA and in the *oszip09* mutant, indicating that OsbZIP09 modulates seed germination via a common set of downstream target genes that are involved in ABA signaling. Phylogenetic analysis and amino-acid sequence comparison indicated that several key domains of bZIP09 were conserved among different plant species, especially the C-terminus that contains a basic DNA-binding region and the adjacent ZIP domain (Figure 2C, Figure S2). This suggests that OsbZIP09 shares a similar DNA binding activity with other bZIP members. We propose that the other non-conserved domains of OsbZIP09 are responsible for its promotive effect on seed germination, which might be mediated by interactions with other regulatory proteins. This deserves further study to identify the OsZIP09 interaction proteins and dissect the underlying mechanisms.

Analysis of PHS and seed germination, combined with RNA-seq and DAP-seq data, indicated that OsbZIP09 regulates seed germination via interacting with ABA signaling to coordinate the expression of common downstream target genes (Figure 1 and Figure 5). Approximately 90% of the 52 direct target genes of OsbZIP09 showed the same change in expression in response to mutation of OsbZIP09 and ABA treatment. Notably, six *LEA* genes were present among the 52 key OsbZIP09 targets and their expression was upregulated in *osbzip09* and ABA-treated rice seeds (Figure 5 and Figure 6, Table 1). LEA proteins accumulate in maturing seeds, and represent a hallmark of seed maturation. The expression of *LEA* genes is induced by drying, freezing, high salinity, osmotic stress, and exogenous ABA [51,52,53]. The accumulation of LEA proteins potentially protects cellular structures and thus strengthens plant tolerance to dehydration [54,55]. Rice contains 34 LEA proteins which have been subdivided into seven groups [56]. LEAs are often considered to be ABA-responsive genes that mediate plant adaptation to various stresses, such as drought stress and antioxidant stress [57,58,59]. Recently, two reports have revealed that LEAs are also involved in the regulation of rice seed germination: the germination of *OsLEA5*-RNAi transgenic rice seeds was less sensitive to ABA treatment [60], and the mutation of *OsLEA33* promoted post-germination growth of rice [25]. These studies suggest that OsLEAs play positive roles in plant stress responses and negative roles in rice seed germination. Here, the expression of six *OsLEA* genes increased in response to the mutation of OsbZIP09 and ABA treatment, which is consistent with the observed decreased PHS and seed germination phenotype in the *oszip09* mutant and ABA-treated wild type. Most importantly, the identified *OsLEA* genes here were all direct targets of OsbZIP09, implying that the transcriptional regulation of *OsLEA* family genes is an important mechanism by which OsbZIP09 modulates seed dormancy and germination. Furthermore, *OsLEA25*, which was selected in this study for qRT-PCR and dual-luciferase validation, was also identified previously in a GA-responsive proteomic assay, in which GA suppressed the accumulation of OsLEA25 and four other OsLEA proteins during seed germination [25]. Therefore, we propose that *OsLEA* family genes play essential roles in phytohormone-regulated seed germination in rice. In addition to *OsLEA25*, an AWPM-19-like family gene and a gene encoding universal stress protein (USP) were also identified in two “omic” studies. Proteomic data indicated that GA-suppressed the expression of AWPM-19 and USP proteins, whereas here, ABA treatment and mutation of *OsbZIP09* enhanced their expression. AWPM-19 family proteins have been identified in several major crops, including wheat, barley, and rice [61,62,63]. Two AWPM-19 family proteins, PM19-A1 and PM19-A2, accumulated more in maturing grains of dormant wheat than in non-dormant genotypes [64]. In rice, knock-down of an *AWPM-19* gene, *OsPM1*, which controls ABA influx, promoted seed germination, whereas *OsPM1* overexpression had the opposite effect [65]. These studies in wheat and rice indicated that AWPM-19 family proteins negatively regulate seed germination. Although only 8 out of the 52 OsZIP09 targets were downregulated in *osbzip09* and ABA-treated seeds, they included two key genes. One was *DLT*, which encodes a transcription factor that mediates the regulation of plant architecture by brassinosteroid, especially plant height and leaf angle [66,67]. Although no evidence currently exists that DLT regulates seed germination, it associates OsbZIP09 and BR signaling in modulating seed germination and post-germination growth. A second gene, *OsLOX2*, encodes a lipoxygenase that degrades storage lipids during rice seed germination. Overexpression of *OsLOX2* accelerates rice seed germination under normal conditions and decreases seed viability after accelerated aging [26]. 

We have shown that mutation of *OsbZIP09* leads to a notable reduction in rice PHS. OsbZIP09 is a typical bZIP transcription factor that contains conserved bZIP domains and its expression is induced by ABA. To reveal the mechanism whereby OsbZIP09 regulates seed germination, RNA-seq and DAP-seq results isolated 52 key direct targets of OsbZIP09, most of which showed consistent changes in expression in response to ABA and in the *osbzip09* mutant. Some of these genes, such as *OsLOX2* and *LEA* genes are involved in controlling seed germination. Therefore, we have successfully identified a number of key direct target genes of OsbZIP09 to explain how OsbZIP09 controls rice seed germination.

## 4. Materials and Methods

### 4.1. Generation of Osbzip09 Mutants Using CRISPR/Cas9

One specific target site within the first exon of *OsbZIP09* was selected for gene editing. The target sequence was introduced into vector pC1300-Cas9, and the generated construct was transformed into japonica rice ZH11 via *Agrobacterium*-mediated transformation. The CRISPR/Cas9 vector system and the detailed procedure have been described previously [68].

### 4.2. Rice Growth Conditions 

All rice plants were grown under the same climatic and management conditions during the summer in a paddy field at Yangzhou University (Yangzhou, China). Three replicate plots were used for the experiments, and the plots were arranged in a randomized block pattern, with 6 rows per plot and 10 plants per row. Superior spikelets or mature seeds from superior spikelets from the middle of each plot were collected for seed germination analysis. 

### 4.3. Seed Germination Analysis

To mimic PHS in the lab, mature panicles from *osbzip09* mutants and ZH11 wild-type controls were collected and immersed in water, and the number of germinated seeds was recorded after six days. Traditional germination assay of rice seeds under normal conditions was performed as described previously [69]. In brief, dehulled rice seeds were sterilized with 70% ethanol and washed twice with Milli-Q water for each experiment. Sterilized seeds were subsequently germinated in darkness in an artificial climate incubator at a temperature of 26 °C and a relative humidity of 70%. Seeds with a radicle longer than 1 mm were considered to have successfully germinated [70]. Germination rates were recorded every 12 h until 120 h after imbibition (HAI). Each seed germination assay included at least three independent biological replicates, and each replicate contained 30 seeds. 

### 4.4. Phylogenetic Assay, Sequence Alignment, and Domain Analysis of bZIP09 

The BLAST function of the National Center for Biotechnology Information (NCBI) database (Bethesda, MD, USA) was used to identify highly homologous bZIP09 protein sequences from different species. A phylogenetic tree was then constructed based on the amino-acid sequence of the full-length protein using MEGA6 software. The amino-acid sequences of bZIP09 orthologs from eight representative species was aligned using Clustal Omega (Hinxton, Cambridge, UK) [71]. The conserved amino-acid sequence of the bZIP09 protein was analyzed and a graphical representation was produced by WebLogo 3 (Berkeley, CA, USA) [72]. Finally, SWISS-MODEL (Basel, Switzerland) was used to analyze the predicated three-dimensional (3D) structure of the conserved domain of bZIP09 [73]. 

### 4.5. RNA Extraction and qRT-PCR Analysis

WT seeds treated with ABA (5 μM) or EtOH (mock treatment) for 15 and 30 min were collected for quantification of *OsbZIP09* expression via qRT-PCR. Seeds of WT treated with ABA (5 μM) or mock and *osbzip09-*2 mutant seeds mock-treated for 36 HAI were collected for RNA-seq and qRT-PCR validation. Three biological replicates were included for each experiment, and *ACTIN* served as a reference gene for normalization. Total RNA was isolated from the harvested seed samples using an RNAsimple Total RNA kit (TIANGEN, Beijing, China), and genomic DNA was removed by treatment with RNase-free DNase I (Qiagen, Hilden, Germany). First-strand cDNA was synthesized using HiScriptIII RT SuperMix for qPCR (Vazyme, Nanjing, China) with an oligo dT primer.

### 4.6. RNA-Seq Analysis

High-quality RNA (1 µg) was used for library generation, and high-throughput RNA sequencing was performed on an Illumina Novaseq platform (San Diego, CA, USA), and 150 bp paired-end reads were generated. Raw sequencing reads were trimmed, and the collected clean data were aligned to the genome of rice japonica cultivar Nipponbare (IRGSP-1.0, http://rapdb.dna.affrc.go.jp/, accessed on 10 September 2020) using TopHat2 software [74]. The DESeq2 R package (version 1.16.1, Heidelberg, Germany) was used for differential expression analysis [75]. Genes identified by DESeq2 with a *p*-value < 0.05 and fold change >1.5 were assigned as being differentially expressed.

### 4.7. DNA Affinity Purification Sequencing (DAP-Seq) Sampling

DAP-seq was performed according to Bartlett et al. (2017) [76]. First, genomic DNA (gDNA) was extracted from mature seeds of rice. Then a gDNA DAP-seq library was prepared by attaching a short DNA sequencing adaptor to the purified and fragmented gDNA. The adapter sequences were truncated Illumina TruSeq adapters; the TruSeq Universal and Index adapters corresponded to the DAP-seq Adapter A and Adapter B. The DAP gDNA library was prepared using a kit from NEBNext^®^ DNA Library Prep Master Mix Set for Illumina^®^ (NEB, #E6040S/L, Ipswich, MA, USA). OsbZIP09 was fused to the HaloTag using a kit from pFN19K HaloTag T7 SP6 Flexi Vecto (Promega #G184A). OsbZIP09 fused to HaloTag was expressed using a TnT SP6 High-Yield Wheat Germ Protein Expression System (L3260, Promega, Madison, WI, USA), and was purified using Magne HaloTag Beads (G7281, Promega). The Magne HaloTag Beads and OsbZIP09-HaloTag mixture were incubated with 500 ng DNA library in 40 μL PBS (Phosphate Buffered Saline) buffer with slow rotation in a cold room for 1.5 h. The beads were washed five times with 200 μL PBS + NP40 (0.005%), resuspended in PBS buffer, the supernatant was removed, and 25 μL EB buffer was added and samples were incubated for 10 min at 98 °C to elute the bound DNA from the beads. The correct DAP-seq library concentration to achieve a specific read count was calculated on the basis of on library fragment size. Negative-control mock DAP-seq libraries were prepared as described above, without the addition of protein to the beads.

### 4.8. DAP-Seq Data Analysis

We defined target genes as those that contained DAP-seq peaks located within the transcribed regions of genes, introns, or 2 kb upstream from the transcription start site (TSS), or 2 kb downstream from the transcription termination site (TTS). DAP-seq reads were aligned to the rice genome using Bowtie 2 (Baltimore, MD, USA) [77], which supports gapped and paired-end alignment modes. We ran Bowtie version 2.2.3 with default parameters and reported only unique alignments. DAP-seq peaks were detected by MACS2 (Boston, MA, USA) [24]. We used MACS version 2.0.10 with default parameters, as duplicates were allowed, and the *q*-value < 0.05. Core motifs were identified by MEME-ChIP (Brisbane, QLD, Australia) [78].

### 4.9. Promoter Analysis

To analyze ABA-responsive motifs within the *OsbZIP09* promoter, 2-kb DNA sequence upstream of the *OsbZIP09* initiation codon ATG was obtained from The Rice Annotation Project Database (Rap-db). The promoter sequence of *OsbZIP09* was scanned in PLACE database to identify the presence of putative ABA-responsive *cis*-acting elements. To identify OsbZIP09-binding motifs within the promoters of its target genes, the 2-kb promoter sequence of each gene was analyzed using *VectorNTI9* software (version 9, Invitrogen, Carlsbad, CA, USA) to confirm the location and number of the motifs identified by DAP-seq. 

### 4.10. Dual-Luciferase Reporter Assay

*Agrobacterium*-mediated transient assays were conducted with tobacco leaves by co-expressing reporter and effector constructs. The promoters of *LEA25* (1.15 kb) and *LOX2* (2.05 kb) were each cloned into the pGreen II0800-LUC vector to generate reporter constructs. The coding region of *OsbZIP09* was cloned into the pGreenII 62-SK vector to generate an effector construct [79]. Each reporter construct was transformed into tobacco leaves together with the OsbZIP09 effector. After 36 h growth, the relative LUC activity of the transformed tobacco leaves with and without ABA (5 μM) treatment were measured using the Dual-Luciferase Reporter Assay System (Vazyme, Nanjing, China). The detailed method is described previously [80]. The sequences of all the primers used in this study are listed in Table S1.

## Figures and Tables

**Table 1:** List of OsbZIP09 target genes upregulated by ABA and in *osbzip09-*2.

Gene ID	Transcriptome Analysis Log_2_(Fold Change) ^1^		DAP-Seq Analysis		Description
	*osbzip09-*2/WT	ABA/Mock (WT)	−Log_10_ (*p* Value)	Distance to TSS (bp) ^2^	
*Os10g0173000*	2.05	3.94	14.30	1616	expressed protein
*Os08g0529300*	2.13	2.52	11.59	95	F-box protein 463
*Os11g0154900*	0.95	1.30	17.17	114	OsZIP-2a; OsbZIP80
*Os03g0386000*	0.82	3.04	12.68	1374	SRWD5
*Os03g0820500*	1.18	1.09	9.65	373	OsADF3
*Os02g0716800*	2.20	0.96	6.91	1543	PTR2
*Os05g0542500*	0.68	1.97	12.53	552	OsLEA3
*Os01g0702500*	0.68	1.42	10.54	3	OsLEA22
*Os05g0563900*	0.87	0.81	10.25	1498	OsGRX17
*Os09g0325700*	0.63	1.53	11.80	37	OsPP108; SIPP2C1
*Os02g0740500*	0.85	0.90	9.65	1299	expressed protein
*Os04g0617050*	0.79	0.84	9.20	110	OsSAUR21
*Os12g0478200*	1.12	1.02	7.73	1383	GRAM protein
*Os05g0198400*	0.97	0.71	11.26	52	OsZIP7a
*Os10g0524300*	1.24	1.89	7.66	1330	EMBRYO SAC 1
*Os09g0484800*	0.84	0.85	9.25	101	pirin, putative
*Os10g0159033*	2.20	1.29	12.19	135	expressed protein
*Os03g0723400*	0.64	2.04	9.57	6	expressed protein
*Os10g0542100*	1.13	2.38	8.48	2	OsMT-II-1a
*Os06g0110200*	0.66	1.20	8.45	62	OsLEA4
*Os04g0385600*	0.77	0.99	10.06	522	OsFBO3
*Os09g0127700*	0.64	1.36	9.25	18	expressed protein
*Os03g0170900*	0.99	0.63	10.24	1082	OsSUT1
*Os11g0454200*	0.95	3.05	8.23	97	OsLEA28
*Os11g0451700*	0.95	2.27	8.77	118	OsLEA25, Rab17
*Os08g0150700*	0.93	0.85	9.88	881	OsFbox407
*Os05g0572700*	0.97	1.49	12.68	745	OsPP2C51
*Os05g0524100*	0.77	1.30	7.79	234	expressed protein
*Os07g0422100*	0.61	2.17	6.17	247	OsPM19L2
*Os06g0697200*	0.74	0.68	7.53	1321	OsRH35B
*Os10g0177200*	0.71	2.29	12.03	856	OsDSR-1
*Os04g0610600*	0.88	1.93	5.95	12	OsLEA18
*Os01g0135700*	0.68	0.98	8.55	155	OsCML16
*Os02g0718600*	0.88	1.55	13.89	388	expressed protein
*Os01g0849600*	1.58	1.73	6.90	70	USP protein
*Os09g0502500*	1.19	0.66	8.12	162	alcohol dehydrogenase
*Os12g0510750*	0.85	1.74	8.45	162	expressed protein
*Os06g0261300*	2.05	1.22	4.79	486	expressed protein

^1^ Log_2_(fold change) indicates the log_2_ fold change in gene transcript abundance between *osbzip09-*2 and WT or ABA-treated WT and mock-treated WT. ^2^ Distance to TSS (transcription start site) indicates the distance of the OsbZIP09 binding peak to the nearest transcription start site.

**Table 2:** List of OsbZIP09 target genes downregulated by ABA and in *osbzip09*-2.

Gene ID	Transcriptome Analysis Log_2_(Fold Change) ^1^		DAP-Seq Analysis		Description
	*osbzip09-*2/WT	ABA/Mock(WT)	−Log_10_ (*p* Value)	Distance To TSS ^2^	
*Os06g0127800*	−0.58	−1.84	24.06	220	DLT; OsGRAS-32
*Os04g0678700*	−0.63	−1.47	15.45	111	OsPORA
*Os06g0112100*	−1.30	−3.33	15.80	673	Nucleoside phosphorylase
*Os02g0769200*	−0.73	−1.28	7.64	435	LYP5, Os-LYP5
*Os10g0104900*	−0.80	−2.22	7.29	701	OsCMT3a
*Os03g0306800*	−0.93	−0.72	6.91	329	Calvin-cycle protein CP12
*Os05g0406800*	−0.61	−1.78	5.47	141	receptor-like protein kinase
*Os03g0738600*	−0.64	−2.49	6.91	209	OsLOX2

^1^ Log_2_(fold change) indicates the log_2_ fold change in gene transcript abundance between *osbzip09-*2 and WT or ABA-treated WT and mock-treated WT. ^2^ Distance to TSS indicates the distance of the OsbZIP09 binding peak to the nearest transcription start site.

**Table 3:** List of OsbZIP09 target genes inconsistently regulated by ABA and in *osbzip09-*2.

Gene ID	Transcriptome Analysis Log_2_(Fold Change)^1^		DAP-Seq Analysis		Description
	*osbzip09-*2/WT	ABA/Mock (WT)	−Log_10_(*p* Value)	Distance To TSS (bp) ^2^	
*Os08g0278900*	0.67	−0.74	12.61	80	MIR domain protein
*Os03g0835150*	0.82	−1.59	7.48	568	expressed protein
*Os02g0226200*	1.17	−0.83	8.95	260	IB hydrolase
*Os04g0678400*	0.86	−0.94	6.91	1566	OsDof-17; OsDof18
*Os03g0377700*	−0.75	0.84	8.07	1375	CSLA5-cellulose synthase
*Os02g0115900*	0.70	−0.72	5.95	22	OsBip1; BiP3

^1^ Log_2_(fold change) indicates the log_2_ of the fold change in gene transcript abundance between *osbzip09-*2 and WT or ABA-treated WT and mock-treated WT. ^2^ Distance to TSS indicates the distance of the OsbZIP09 binding peak to the nearest transcription start site.

## Supplementary Materials

The following are available online at https://www.mdpi.com/1422-0067/22/4/1661/s1.

## Abbreviations

ABA	abscisic acid
ABI5	ABA-INSENSITIVE 5
ABFs	ABRE-binding factors
ABRE	ABA-responsive element
AOC	Allene Oxide Cyclase
AREBs	ABA-responsive element binding proteins
AWPM-19	ABA-induced Wheat Plasma Membrane Polypeptide-19
ANOVA	analysis of variance
bZIP	basic leucine zipper
ChIP-seq	Chromatin immunoprecipitation sequencing
CNX	cofactor for nitrate reductase and xanthine dehydrogenase
CSLA5	cellulose synthase-like A5
DAP-seq	DNA affinity purification sequencing
DOG1	DELAY OF GERMINATION 1
DRE	drought-responsive element
HAI	hours after imbibition
DEGs	differentially expressed genes
DLT	DWARF AND LOW-TILLERING
GA	gibberellin
GAP	GTPase activating protein
GO	gene ontology
IPA1	Ideal Plant Architecture 1
KEGG	Kyoto Encyclopedia of Genes and Genomes
LEA	Late Embryogenesis Abundant
LOX2	lipoxygenase 2
MFT2	MOTHER OF FT AND TFL 2
NCBI	National Center for Biotechnology Information
PHS	preharvest sprouting
PLA3	PLASTOCHRON3
PP2Cs	protein phosphatases 2C
PUFAs	polyunsaturated fatty acids
qRT-PCR	quantitative real-time polymerase chain reaction
QTLs	quantitative trait loci
RCAR1	regulatory component of ABA receptor 1
ROS	reactive oxygen species
RNA-Seq	RNA sequencing
SD	standard deviation
SnRK2	sucrose nonfermenting 1-related protein kinase 2
TF	transcription factor
TSS	transcription start site
TTS	transcription termination site
USP	universal stress protein

## References

[1] Shu K., Liu X.D., Xie Q., He Z.H. (2016). Two faces of one seed: Hormonal regulation of dormancy and germination. Mol. Plant.

[2] Finkelstein R., Reeves W., Ariizumi T., Steber C. (2008). Molecular aspects of seed dormancy. Annu. Rev. Plant Biol..

[3] Wang J., Deng Q., Li Y., Yu Y., Liu X., Han Y., Luo X., Wu X., Ju L., Sun J. (2020). Transcription factors Rc and OsVP 1 coordinately regulate preharvest sprouting tolerance in red pericarp rice. J. Agric. Food Chem..

[4] Suzuki Y., Miura K., Shigemune A., Sasahara H., Ohta H., Uehara Y., Ishikawa T., Hamada S., Shirasawa K. (2015). Marker-assisted breeding of a LOX-3-null rice line with improved storability and resistance to preharvest sprouting. Theor. Appl. Genet..

[5] Liu X., Wang J., Yu Y., Kong L., Liu Y., Liu Z., Li H., Wei P., Liu M., Zhou H. (2019). Identification and characterization of the rice pre-harvest sprouting mutants involved in molybdenum cofactor biosynthesis. New Phytol..

[6] Nonogaki H. (2019). Seed germination and dormancy: The classic story, new puzzles, and evolution. J. Integr. Plant Biol..

[7] Yan A., Chen Z. (2017). The pivotal role of abscisic acid signaling during transition from seed maturation to germination. Plant Cell Rep..

[8] Née G., Xiang Y., Soppe W.J. (2017). The release of dormancy, a wake–up call for seeds to germinate. Curr. Opin. Plant Biol..

[9] Martínez-Andújar C., Ordiz M.I., Huang Z., Nonogaki M., Beachy R.N., Nonogaki H. (2011). Induction of 9-cis-epoxycarotenoid dioxygenase in Arabidopsis thaliana seeds enhances seed dormancy. Proc. Natl. Acad. Sci. USA.

[10] Nonogaki M., Sall K., Nambara E., Nonogaki H. (2014). Amplification of ABA biosynthesis and signaling through a positive feedback mechanism in seeds. Plant J..

[11] Chen K., Li G.J., Bressan R.A., Song C.P., Zhu J.K., Zhao Y. (2020). Abscisic acid dynamics, signaling, and functions in plants. J. Integr. Plant Biol..

[12] Miyakawa T., Fujita Y., Yamaguchi–Shinozaki K., Tanokura M. (2013). Structure and function of abscisic acid receptors. Trends Plant Sci..

[13] Zhao H., Nie K., Zhou H., Yan X., Zhan Q., Zheng Y., Song C.P. (2020). ABI5 modulates seed germination via feedback regulation of the expression of the PYR/PYL/RCAR ABA receptor genes. New Phytol..

[14] Deppmann C.D., Alvania R.S., Taparowsky E.J. (2006). Cross-species annotation of basic leucine zipper factor interactions: Insight into the evolution of closed interaction networks. Mol. Biol. Evol..

[15] Corrêa L.G.G., Riaño-Pachón D.M., Schrago C.G., dos Santos R.V., Mueller-Roeber B., Vincentz M. (2008). The role of bZIP transcription factors in green plant evolution: Adaptive features emerging from four founder genes. PLoS ONE.

[16] Dröge-Laser W., Snoek B.L., Snel B., Weiste C. (2018). The Arabidopsis bZIP transcription factor family-an update. Curr. Opin. Plant Biol..

[17] Nijhawan A., Jain M., Tyagi A.K., Khurana J.P. (2008). Genomic survey and gene expression analysis of the basic leucine zipper transcription factor family in rice. Plant Physiol..

[18] Liu X., Chu Z. (2015). Genome-wide evolutionary characterization and analysis of bZIP transcription factors and their expression profiles in response to multiple abiotic stresses in Brachypodium distachyon. BMC Genom..

[19] Liao Y., Zou H.F., Wei W., Hao Y.J., Tian A.G., Huang J., Liu Y.F., Zhang J.S., Chen S.Y. (2008). Soybean GmbZIP44 GmbZIP62 and GmbZIP78 genes function as negative regulator of ABA signaling and confer salt and freezing tolerance in transgenic Arabidopsis. Planta.

[20] Zhou Y., Xu D., Jia L., Huang X., Ma G., Wang S., Zhu M., Zhang A., Guan M., Lu K. (2017). Genome-wide identification and structural analysis of bZIP transcription factor genes in *Brassica napus*. Genes.

[21] Johnson D.S., Mortazavi A., Myers R.M., Wold B. (2007). Genome-wide mapping of in vivo protein-DNA interactions. Science.

[22] O’Malley R.C., Huang S.C., Song L., Lewsey M.G., Bartlett A., Nery J.R., Galli M., Gallavotti A., Ecker J.R. (2016). Cistrome and epicistrome features shape the regulatory DNA landscape. Cell.

[23] Muro-Villanueva F., Mao X., Chapple C. (2019). Linking phenylpropanoid metabolism, lignin deposition, and plant growth inhibition. Curr. Opin. Biotechnol..

[24] Zhang Y., Liu T., Meyer C.A., Eeckhoute J., Johnson D.S., Bernstein B.E., Nusbaum C., Myers R.M., Brown M., Li W. (2008). Model-based analysis of ChIP-Seq (MACS). Genome Biol..

[25] Li Q.F., Zhou Y., Xiong M., Ren X.Y., Han L., Wang J.D., Zhang C.Q., Fan X.L., Liu Q.Q. (2020). Gibberellin recovers seed germination in rice with impaired brassinosteroid signalling. Plant Sci..

[26] Huang J., Cai M., Long Q., Liu L., Lin Q., Jiang L., Chen S., Wan J. (2014). OsLOX2, a rice type I lipoxygenase, confers opposite effects on seed germination and longevity. Transgenic Res..

[27] Fang J., Chai C., Qian Q., Li C., Tang J., Sun L., Huang Z., Guo X., Sun C., Liu M. (2008). Mutations of genes in synthesis of the carotenoid precursors of ABA lead to pre-harvest sprouting and photo-oxidation in rice. Plant J..

[28] Shu K., Meng Y.J., Shuai H.W., Liu W.G., Du J.B., Liu L., Yang W.Y. (2015). Dormancy and germination: How does the crop seed decide?. Plant Biol..

[29] Yang L., Liu S., Lin R. (2020). The role of light in regulating seed dormancy and germination. J. Integr. Plant Biol..

[30] Guo L., Zhu L., Xu Y., Zeng D., Wu P., Qian Q. (2004). QTL analysis of seed dormancy in rice. Euphytica.

[31] Gao F.Y., Ren G.J., Lu X.J., Sun S.X., Li H.J., Gao Y.M., Luo H., Yan W.G., Zhang Y.Z. (2008). QTL analysis for resistance to preharvest sprouting in rice (*Oryza sativa*). Plant Breed..

[32] Ye H., Beighley D.H., Feng J., Gu X.Y. (2013). Genetic and physiological characterization of two clusters of quantitative trait loci associated with seed dormancy and plant height in rice. G3.

[33] Lee G.A., Jeon Y.A., Lee H.S., Hyun D.Y., Lee J.R., Lee M.C., Lee S.Y., Ma K.H., Koh H.J. (2017). New genetic loci associated with preharvest sprouting and its evaluation based on the model equation in rice. Front. Plant Sci..

[34] Cheon K.S., Won Y.J., Jeong Y.M., Lee Y.Y., Kang D.Y., Oh J., Oh H., Kim S.L., Kim N., Lee E. (2020). QTL mapping for pre-harvest sprouting resistance in japonica rice varieties utilizing genome re-sequencing. Mol. Genet. Genom..

[35] Agrawal G.K., Yamazaki M., Kobayashi M., Hirochika R., Miyao A., Hirochika H. (2001). Screening of the rice viviparous mutants generated by endogenous retrotransposon Tos17 insertion. Tagging of a zeaxanthin epoxidase gene and a novel OsTATC gene. Plant Physiol..

[36] Kawakatsu T., Taramino G., Itoh J., Allen J., Sato Y., Hong S.K., Yule R., Nagasawa N., Kojima M., Kusaba M. (2009). PLASTOCHRON3/GOLIATH encodes a glutamate carboxypeptidase required for proper development in rice. Plant J..

[37] Du L., Xu F., Fang J., Gao S., Tang J., Fang S., Wang H., Tong H., Zhang F., Chu J. (2018). Endosperm sugar accumulation caused by mutation of PHS8/ISA1 leads to pre-harvest sprouting in rice. Plant J..

[38] Xu F., Tang J., Gao S., Cheng X., Du L., Chu C. (2019). Control of rice pre-harvest sprouting by glutaredoxin-mediated abscisic acid signaling. Plant J..

[39] Miao C., Wang Z., Zhang L., Yao J., Hua K., Liu X., Shi H., Zhu J.K. (2019). The grain yield modulator miR156 regulates seed dormancy through the gibberellin pathway in rice. Nat. Commun..

[40] Bi C., Ma Y., Wu Z., Yu Y.T., Liang S., Lu K., Wang X.F. (2017). Arabidopsis ABI5 plays a role in regulating ROS homeostasis by activating CATALASE 1 transcription in seed germination. Plant Mol. Biol..

[41] Bai M., Sun J., Liu J., Ren H., Wang K., Wang Y., Wang C., Dehesh K. (2019). The B-box protein BBX19 suppresses seed germination via induction of ABI5. Plant J..

[42] Zou M., Guan Y., Ren H., Zhang F., Chen F. (2008). A bZIP transcription factor, OsABI5, is involved in rice fertility and stress tolerance. Plant Mol. Biol..

[43] Ju L., Jing Y., Shi P., Liu J., Chen J., Yan J., Chu J., Chen K.M., Sun J. (2019). JAZ proteins modulate seed germination through interaction with ABI5 in bread wheat and Arabidopsis. New Phytol..

[44] Utsugi S., Ashikawa I., Nakamura S., Shibasaka M. (2020). TaABI5, a wheat homolog of Arabidopsis thaliana ABA insensitive 5, controls seed germination. J. Plant Res..

[45] Collin A., Daszkowska-Golec A., Kurowska M., Szarejko I. (2020). Barley ABI5 (Abscisic Acid INSENSITIVE 5) is involved in abscisic acid-dependent drought response. Front. Plant Sci..

[46] Cantoro R., Crocco C.D., Benech-Arnold R.L., Rodríguez M.V. (2013). In vitro binding of Sorghum bicolor transcription factors ABI4 and ABI5 to a conserved region of a GA 2-OXIDASE promoter: Possible role of this interaction in the expression of seed dormancy. J. Exp. Bot..

[47] Hossain M.A., Cho J.I., Han M., Ahn C.H., Jeon J.S., An G., Park P.B. (2010). The ABRE–binding bZIP transcription factor OsABF2 is a positive regulator of abiotic stress and ABA signaling in rice. J. Plant. Physiol..

[48] Song S., Wang G., Wu H., Fan X., Liang L., Zhao H., Li S., Hu Y., Liu H., Ayaad M. (2020). OsMFT2 is involved in the regulation of ABA signaling mediated seed germination through interacting with OsbZIP23/66/72 in rice. Plant J..

[49] Wang Y., Hou Y., Qiu J., Wang H., Wang S., Tang L., Tong X., Zhang J. (2020). Abscisic acid promotes jasmonic acid biosynthesis via a ‘SAPK10–bZIP72–AOC’ pathway to synergistically inhibit seed germination in rice (*Oryza sativa*). New Phytol..

[50] Wang Q., Lin Q., Wu T., Duan E., Huang Y., Yang C., Mou C., Lan J., Zhou C., Xie K. (2020). OsDOG1L–3 regulates seed dormancy through the abscisic acid pathway in rice. Plant Sci..

[51] Wise M.J., Tunnacliffe A. (2004). POPP the question: What do LEA proteins do?. Trends Plant Sci..

[52] Cao J., Li X. (2015). Identification and phylogenetic analysis of late embryogenesis abundant proteins family in tomato (*Solanum lycopersicum*). Planta.

[53] Liu Y., Song Q., Li D., Yang X., Li D. (2017). Multifunctional roles of plant dehydrins in response to environmental stresses. Front. Plant Sci..

[54] Yang W., Zhang L., Lv H., Li H., Zhang Y., Xu Y., Yu J. (2015). The K-segments of wheat dehydrin WZY2 are essential for its protective functions under temperature stress. Front. Plant Sci..

[55] Hara M., Monna S., Murata T., Nakano T., Amano S., Nachbar M., Wätzig H. (2016). The Arabidopsis KS-type dehydrin recovers lactate dehydrogenase activity inhibited by copper with the contribution of his residues. Plant Sci..

[56] Wang X.S., Zhu H.B., Jin G.L., Liu H.L., Wu W.R., Zhu J. (2007). Genome-scale identification and analysis of LEA genes in rice (*Oryza sativa* L.). Plant Sci..

[57] Xiao B., Huang Y., Tang N., Xiong L. (2007). Over-expression of a LEA gene in rice improves drought resistance under the field conditions. Theor. Appl. Genet..

[58] Huang L., Zhang M., Jia J., Zhao X., Huang X., Ji E., Ni L., Jiang M. (2018). An atypical late embryogenesis abundant protein OsLEA5 plays a positive role in ABA-induced antioxidant defense in *oryza sativa* L.. Plant Cell Physiol..

[59] Hu T., Liu Y., Zhu S., Qin J., Li W., Zhou N. (2019). Overexpression of OsLea14-A improves the tolerance of rice and increases Hg accumulation under diverse stresses. Environ. Sci. Pollut. Res. Int..

[60] Huang L., Jia J., Zhao X., Zhang M., Huang X., Ji E., Ni L., Jiang M. (2018). The ascorbate peroxidase APX1 is a direct target of a zinc finger transcription factor ZFP36 and a late embryogenesis abundant protein OsLEA5 interacts with ZFP36 to co-regulate OsAPX1 in seed germination in rice. Biochem. Biophys. Res. Commun..

[61] Koike M., Takezawa D., Arakawa K., Yoshida S. (1997). Accumulation of 19-kDa plasma membrane polypeptide during induction of freezing tolerance in wheat suspensioncultured cells by abscisic acid. Plant Cell Physiol..

[62] Ranford J.C., Bryce J.H., Morris P.C. (2002). PM19, a barley (*Hordeum vulgare* L.) gene encoding a putative plasma membrane protein, is expressed during embryo development and dormancy. J. Exp. Bot..

[63] Chen H., Lan H., Huang P., Zhang Y., Yuan X., Huang X., Huang J., Zhang H. (2015). Characterization of OsPM19L1 encoding an AWPM-19-like family protein that is dramatically induced by osmotic stress in rice. Genet. Mol. Res..

[64] Barrero J.M., Cavanagh C., Verbyla K.L., Tibbits J.F., Verbyla A.P., Huang B.E., Rosewarne G.M., Stephen S., Wang P., Whan A. (2015). Transcriptomic analysis of wheat nearisogenic lines identifies PM19-A1 and A2 as candidates for a major dormancy QTL. Genome Biol..

[65] Yao L., Cheng X., Gu Z., Huang W., Li S., Wang L., Wang Y.F., Xu P., Ma H., Ge X. (2018). The AWPM-19 family protein OsPM1 mediates abscisic acid influx and drought response in rice. Plant Cell.

[66] Tong H., Liu L., Jin Y., Du L., Yin Y., Qian Q., Zhu L., Chu C. (2012). Dwarf and low-tillering acts as a direct downstream target of a GSK3/SHAGGY-like kinase to mediate brassinosteroid responses in rice. Plant Cell.

[67] Hirano K., Yoshida H., Aya K., Kawamura M., Hayashi M., Hobo T., Sato-Izawa K., Kitano H., Ueguchi-Tanaka M., Matsuoka M. (2017). Small organ size 1 and small organ size 2/dwarf and low-tillering form a complex to integrate auxin and brassinosteroid signaling in rice. Mol. Plant.

[68] Wang C., Liu Q., Shen Y., Hua Y.F., Wang J.J., Lin J.R., Wu M.G., Sun T.T., Cheng Z.K., Mercier R. (2019). Clonal seeds from hybrid rice by simultaneous genome engineering of meiosis and fertilization genes. Nat. Biotechnol..

[69] Li Q.F., Xiong M., Xu P., Huang L.C., Zhang C.Q., Liu Q.Q. (2016). Dissection of brassinosteroid-regulated proteins in rice embryos during germination by quantitative proteomics. Sci. Rep..

[70] Cheng X.Y., Wu Y., Guo J.P., Du B., Chen R.Z., Zhu L.L., He G.C. (2013). A rice lectin receptor-like kinase that is involved in innate immune responses also contributes to seed germination. Plant J..

[71] Madeira F., Park Y.M., Lee J., Buso N., Gur T., Madhusoodanan N., Basutkar P., Tivey A.R.N., Potter S.C., Finn R.D. (2019). The EMBL-EBI search and sequence analysis tools APIs in 2019. Nucleic Acids Res..

[72] Crooks G.E., Hon G., Chandonia J.M., Brenner S.E. (2004). WebLogo: A sequence logo generator. Genome Res..

[73] Waterhouse A., Bertoni M., Bienert S., Studer G., Tauriello G., Gumienny R., Heer F.T., de Beer T.A.P., Rempfer C., Bordoli L. (2018). SWISS-MODEL: Homology modelling of protein structures and complexes. Nucleic Acids Res..

[74] Kim D., Pertea G., Trapnell C., Pimentel H., Kelley R., Salzberg S.L. (2013). TopHat2: Accurate alignment of transcriptomes in the presence of insertions, deletions and gene fusions. Genome Biol..

[75] Love M.I., Huber W., Anders S. (2014). Moderated estimation of fold change and dispersion for RNA-seq data with DESeq2. Genome Biol..

[76] Bartlett A., O’Malley R.C., Huang S.C., Galli M., Nery J.R., Gallavotti A., Ecker J.R. (2017). Mapping genome-wide transcription-factor binding sites using DAP-seq. Nat. Protoc..

[77] Langmead B., Salzberg S.L. (2012). Fast gapped-read alignment with Bowtie 2. Nat. Methods.

[78] Machanick P., Bailey T.L. (2011). MEME-ChIP: Motif analysis of large DNA datasets. Bioinformatics.

[79] Hellens R.P., Allan A.C., Friel E.N., Bolitho K., Grafton K., Templeton M.D., Karunairetnam S., Gleave A.P., Laing W.A. (2005). Transient expression vectors for functional genomics, quantification of promoter activity and RNA silencing in plants. Plant Methods.

[80] Li Q.F., He J.X. (2016). BZR1 interacts with HY5 to mediate brassinosteroid- and light-regulated cotyledon opening in Arabidopsis in darkness. Mol. Plant.

