# Supplementary material for: Genome-Wide Identification and Expression Analysis of OsbZIP09 Target Genes in Rice Reveal Its Mechanism of Controlling Seed Germination
Source: Int J Mol Sci. 2021 Feb 7;22(4):1661. doi: 10.3390/ijms22041661 (PMC7915905; doi:10.3390/ijms22041661)
Supplement: Supplementary file 1 [file ijms-22-01661-s001.pdf]

Supplementary Material

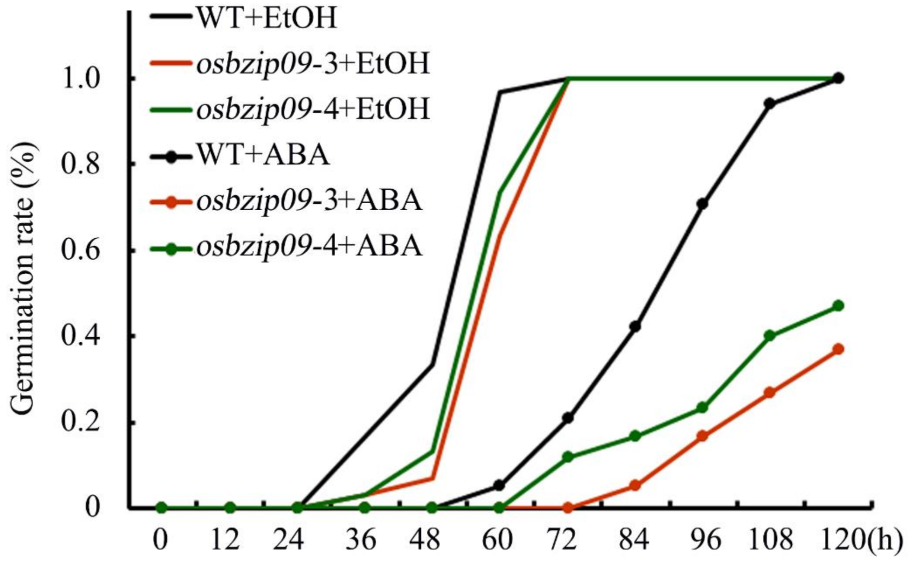

Figure S1. Time-course analysis of seed germination of *osbzip09* mutants (*osbzip09-3* and *osbzip09-4*) under normal conditions.

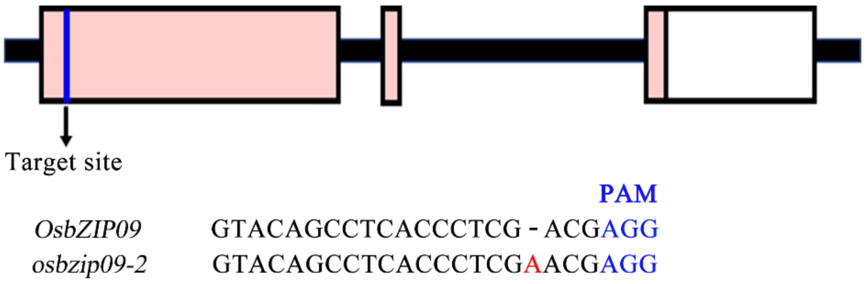

Figure S2. Schematic diagram of the CRISPR/Cas9 target site in Osb*ZIP09* and the basis of the mutation in *osbzip09-2*. The protospacer adjacent motif (PAM) is highlighted in blue.

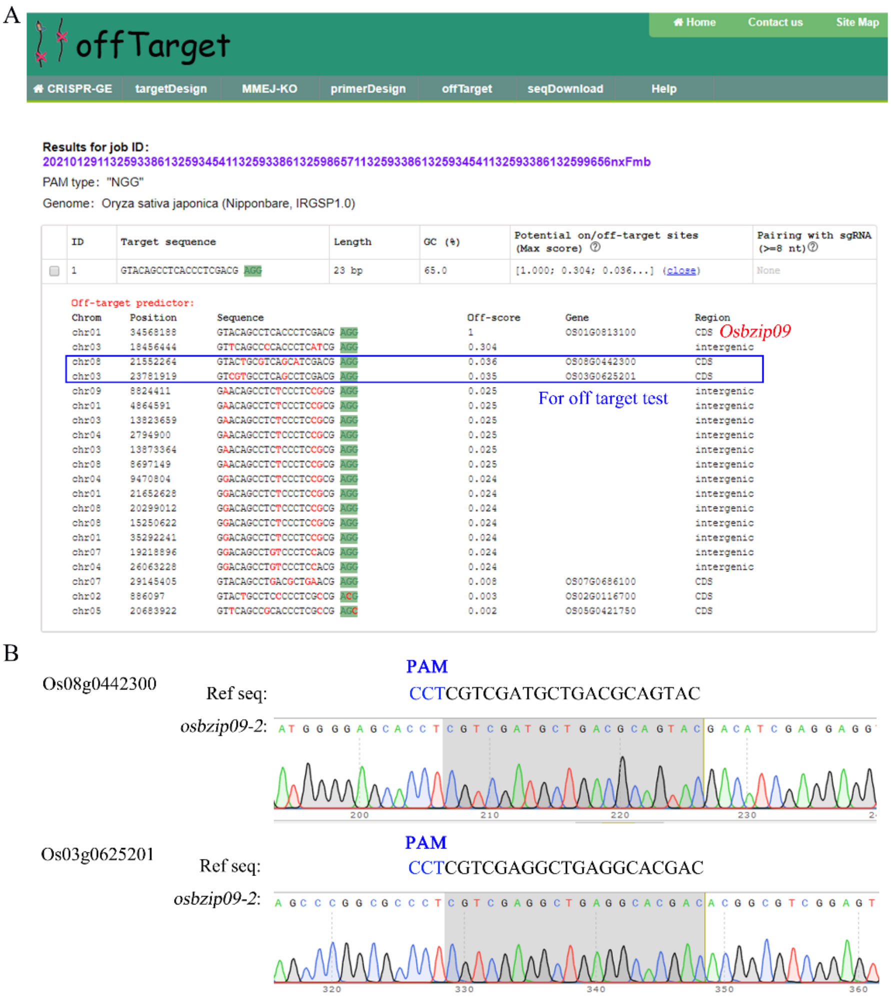

Figure S3. Off target analysis of *osbzip09-2* mutant. (A) Off target analysis of *OsbZIP09* target sequence for CRISPR/Cas9 mediated gene editing using CRISPR-GE (http://skl.scau.edu.cn/home/). (B) Sequencing result of two candidate genes with the highest off-scores indicated that there was no off target mutation in *osbzip09-2* mutant.

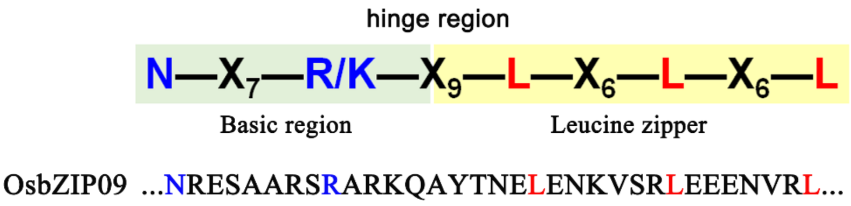

Figure S4. Schematic representation of the bZIP domain and the corresponding amino-acid sequences in OsbZIP09. The sterotypical bZIP domain consists of a basic DNA-binding region (green) and an adjacent ZIP domain (yellow).

A

Preprocessing results of sequencing data quality

sample	raw_reads	clean_reads	clean_bases	error_rate	Q30
WT_EtOH1	44074686	42961196	6.44G	0.03	89.58
WT_EtOH2	49979658	48698646	7.3G	0.03	89.46
WT_EtOH3	41776944	40838000	6.13G	0.03	89.66
WT_ABA1	43466480	42567554	6.39G	0.03	89.13
WT_ABA2	44395760	43421160	6.51G	0.03	90.45
WT_ABA3	45528046	44457036	6.67G	0.03	90.52
Mu_EtOH1	42927938	41912434	6.29G	0.03	90.01
Mu_EtOH2	43224100	42023958	6.3G	0.03	90.06
Mu_EtOH3	43330198	42375872	6.36G	0.03	89.82

B

Mapping results of RNA reads

sample	total_reads	total_map	unique_map	multi_map	proper_map
WT_EtOH1	42961196	39225159(91.3%)	37921255(88.27%)	1303904(3.04%)	34873664(81.17%)
WT_EtOH2	48698646	44429467(91.23%)	42989478(88.28%)	1439989(2.96%)	39466048(81.04%)
WT_EtOH3	40838000	37336878(91.43%)	36084926(88.36%)	1251952(3.07%)	33286780(81.51%)
WT_ABA1	42567554	38695868(90.9%)	37433734(87.94%)	1262134(2.97%)	34278504(80.53%)
WT_ABA2	43421160	40290562(92.79%)	38955087(89.71%)	1335475(3.08%)	36441112(83.92%)
WT_ABA3	44457036	41266915(92.82%)	40018949(90.02%)	1247966(2.81%)	37415444(84.16%)
Mu_EtOH1	41912434	38495520(91.85%)	37301396(89.0%)	1194124(2.85%)	34589228(82.53%)
Mu_EtOH2	42023958	38669044(92.02%)	37509629(89.26%)	1159415(2.76%)	34798630(82.81%)
Mu_EtOH3	42375872	38825789(91.62%)	37564619(88.65%)	1261170(2.98%)	34682192(81.84%)

C

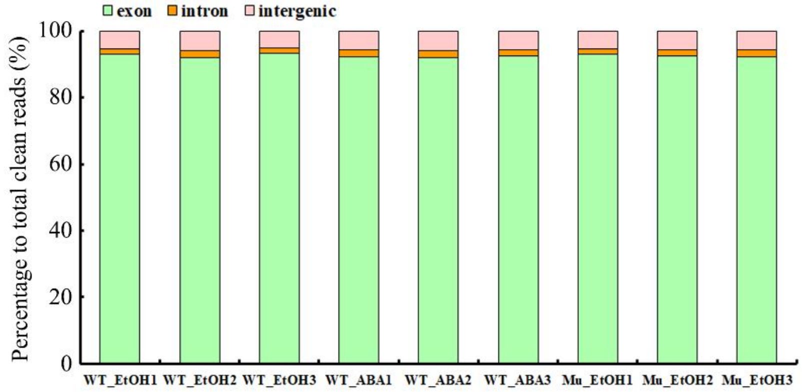

Figure S5. Quality analyses of the RNA-seq data. (A) Preprocessing results of sequencing data quality in wild-type ZH11 treated with ABA or mock-treated, and *osbzip09* treated with mock (n = 3). (B) Mapping result of RNA reads of RNA-seq data. (C) Analysis of the read distribution in each sample.

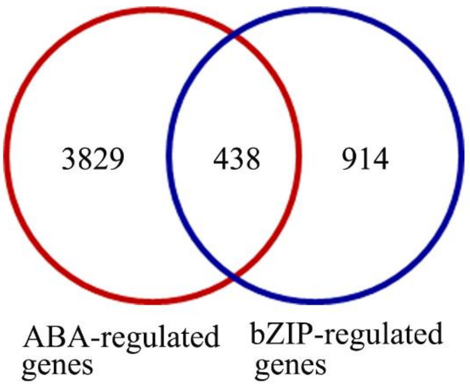

Figure S6. Venn diagram showing the overlap between genes responsive to ABA and mutation of *OsbZIP09*.

Table S1 Oligonucleotide primer sequences used in this study

Primer Names	Sequence (5' → 3')	Usage
OsbZIP09-SG-seq	GTACAGCCTCACCCTCGACG	SG sequence for editing *OsbZIP09*
OsbZIP09seq-F	ATCTCGTCTCGATCATGGGG	Primers for sequencing and confirming mutation of *OsbZIP09*
OsbZIP09seq-R	CCCCAGCTTTGACCAGGAAA	
bZIP09qRT-F	GAGGCATGTCAGGGGATGTG	For qRT-PCR analysis of *OsbZIP09*
bZIP09qRT-R	ACTTGGGTTCTGGTACAGGC	
LEA3qRT-F	TCACTTCAAATTCGGTGCAA	For qRT-PCR analysis of *LEA3*
LEA3qRT-R	CACACCCGTCAGAAATCCTC	
LEA4qRT-F	CAGCACAGGCTCCATAAGCA	For qRT-PCR analysis of *LEA4*
LEA4qRT-R	CAGCACAGAGAGACATGCGT	
LEA18qRT-F	TCCCATCCATGTCCAGGTTGA	For qRT-PCR analysis of *LEA18*
LEA18qRT-R	ACGGCCTCGGATGATGAAGC	
LEA25qRT-F	AGCTGGTGGACTAGGAACCG	For qRT-PCR analysis of *LEA25*
LEA25qRT-R	ATGGTGTCGGGTGTTGTGG	
PP2C51RT-F	TGACGAGTTGGAACGAGTGG	For qRT-PCR analysis of *PP2C51*
PP2C51RT-R	CTCGCCAGGATCAGGAACTC	
USPqRT-F	GTCCGTGGTGCTGAAGATGT	For qRT-PCR analysis of *USP*
USPqRT-R	GCGACTACTTTGCCTTGACG	
LOX2qRT-F	AAGAACGCCCTCGACATCAA	For qRT-PCR analysis of *LOX2*
LOX2qRT-R	CGAACTTGGACTCTCCCGTC	
CSLA5qRT-F	CACCGCCCGGAGAAACAA	For qRT-PCR analysis of *CSLA5*

CSLA5qRT-R	CACCACCAGCATCACCGACAT	
Actin1qRT-F	CCAAGGCCAATCGTGAGAAGA	Reference gene for qRT-PCR assay
Actin1qRT-R	AATCAGTGAGATCACGCCCAG	
pZIP09-EcoRI-F	CGGAATTCATGGCGTCGAAGGCCGGAG	For construction of *OsbZIP09-62-SK*
pZIP09-KpnI-R	GGGGTACCTCAGAAATCTGCGGAGCTTG	
proLEA25KpnI-F	GGGGTACCTCCTTGCTTTTCTCGTTTGA	For construction of *LEA25pro-pGreenII*
proLEA25BamH-R	CGGGATCCTGTGGTGGGTGAAGCGAGGA	
proLOX2KpnI-F	GGGGTACCAGCACGTTTTTCAACGCCC	For construction of *LOX2pro-pGreenII*
proLOX2NcoI-R	CATGCCATGGTCTCTCACTAACCAACCAATGCT	
DAPseq Adapter A	CACGACGCTCTTCCGATCT	For DAP-seq sampling
DAPseq Adapter B	GATCGGAAGAGCACACGTCTG	
